# Demonstration of Complementary Ternary Graphene Field-Effect Transistors

**DOI:** 10.1038/srep39353

**Published:** 2016-12-19

**Authors:** Yun Ji Kim, So-Young Kim, Jinwoo Noh, Chang Hoo Shim, Ukjin Jung, Sang Kyung Lee, Kyoung Eun Chang, Chunhum Cho, Byoung Hun Lee

**Affiliations:** 1 Center for Emerging Electronic Devices and Systems, School of Materials Science and Engineering, Gwangju Institute of Science and Technology, Oryong-dong 1, Buk-gu, Gwangju, 500-712, Korea; 2 Department of Nanobio Materials and Electronics, Gwangju Institute of Science and Technology, Oryong-dong 1, Buk-gu, Gwangju, 500-712, Korea

## Abstract

Strong demand for power reduction in state-of-the-art semiconductor devices calls for novel devices and architectures. Since ternary logic architecture can perform the same function as binary logic architecture with a much lower device density and higher information density, a switch device suitable for the ternary logic has been pursued for several decades. However, a single device that satisfies all the requirements for ternary logic architecture has not been demonstrated. We demonstrated a ternary graphene field-effect transistor (TGFET), showing three discrete current states in one device. The ternary function was achieved by introducing a metal strip to the middle of graphene channel, which created an N-P-N or P-N-P doping pattern depending on the work function of the metal. In addition, a standard ternary inverter working at room temperature has been achieved by modulating the work function of the metal in a graphene channel. The feasibility of a ternary inverter indicates that a general ternary logic architecture can be realized using complementary TGFETs. This breakthrough will provide a key stepping-stone for an extreme-low-power computing technology.

According to Moore’s law, the number of transistors in an integrated circuit doubles approximately every two years. The channel length and gate oxide thickness of field-effect transistors (FETs) should decrease accordingly, but the scaling of these parameters creates many challenges such as increases in the gate leakage current, process cost, and system power, and a degradation of reliability^1^,^2^,^3^. Therefore, there has been a strong demand for novel devices and architectures that can drastically reduce the power consumption in high-performance computing systems.

At the device level, a number of novel devices have been explored, including carbon nanotube (CNT) FETs, graphene FETs, nanowire FETs, ferroelectric FETs, tunnel FETs, nanoelectromechanical systems (NEMS), single-electron transistors (SETs), and transition metal dichalcogenide (TMD)-material-based FETs^4^,^5^,^6^,^7^,^8^,^9^,^10^. At the architecture level, starting with monolithic 3D architecture, many new architectures such as neuromorphic architecture, reconfigurable logic architecture, logic-memory hybrid architecture, multivalued logic architecture are being investigated^11^,^12^,^13^,^14^,^15^,^16^. However, none of the above devices or architecture options have been accepted as a dominant technical option for post-silicon and post von Neumann technology.

Although multivalued logic architecture may be the least popular option among the abovementioned technologies, a multivalued logic architecture—more specifically, a ternary logic architecture—has been investigated for more than six decades. The first ternary computer, SETUN, was commercialized in 1958^17^,^18^. SETUN used only 60% of the vacuum tubes in a binary system because the circuits could be simplified using ternary logic architecture^19^,^20^. However, the binary logic architecture has dominated computing technology since the 1970 s because there was no ternary device that could perform a ternary logic at a single-device level. Since then, numerous electronic devices have been explored to realize a simple ternary architecture or further multivalued logic. These include resonant tunneling diodes, resonant tunneling transistors, neuron MOS transistors, SETs, CNT FETs, and quantum dot FETs^16^,^20^,^21^,^22^,^23^,^24^,^25^,^26^. Resonant tunneling diode had to use multiple devices to generate multiple states, and the fabrication processes using compound semiconductors were too complex to be competitive with silicon technology^22^,^23^. Ternary logic using SET was only functional at cryogenic temperatures, and the separation of logic states was only a few tens of mV^9^,^16^. Quantum dot FETs exhibited three states at room temperature, but the scalability and stability of the devices were limited by the size of the quantum dots and the reliability of the gate dielectric^20^,^21^. So far, the search for a single-device that performs ternary logic has not been successful even though the general architecture is already in place.

An ideal ternary switch should have distinctly separated multiple states within a given operational bias range. For low-power operation, the operational bias should be low enough, for example lower than 1 V. In addition, it is preferred to use an intrinsic mechanism to generate multiple device states rather than combining multiple devices to maximize power efficiency and achieve system scalability. For example, it is possible to generate different states by applying different biases to a silicon MOSFET. However, the variability between the ON and OFF state of a silicon MOSFET will be too high because the thermal emission rate of electrons overcoming the energy barrier at the source side changes exponentially as a function of gate bias.

In this sense, graphene is an ideal material for the ternary device function because the conductivity of graphene is linearly proportional to the gate bias. Intrinsic material properties of graphene, such as a zero bandgap and the density of the state being linearly proportional to the external bias, provide this unique opportunity. Thus, the conductivity of a graphene channel can be engineered to have a stepwise function by making a small region of opposite charge carriers in a graphene channel. For example, if a positive gate bias applied to a graphene FET with a channel having an N-P-N doping profile is progressively increased, the channel doping will be eventually changed to N-N-N doping profile. When the P-type region is changed to N-type by the gate bias, the Fermi level moves through the charge neutrality point where the density of state is zero, and then the resistance of that portion of the graphene channel increases rapidly. Because of this increase in the resistance, the transfer curve of graphene EFT shows flat or decreasing shape. Because of this phenomenon, stepwise current-voltage characteristics can be achieved.

To realize this device, a stable method to shift the Fermi level of graphene into an electron or hole branch is necessary. Various kinds of doping process for graphene have been reported, including chemical doping, metal contact, self-assembled monolayer, and electrical doping^27^,^28^,^29^,^30^. Among these methods, the doping method using metals with different work functions is adopted in this work because this approach is thermally stable, and it is easy to control the area of doping. Theoretically, when graphene is in contact with a low- (or high-) work function metal, it should be doped with an electron (or hole)^31^. For example, an Al strip in contact with graphene will move the Fermi level of graphene from 4.5 eV to 4.08 eV, and a Pt strip will move the Fermi level of graphene toward 5.35 eV. However, it has been difficult to obtain p-type graphene using a metal contact because of a phenomenon similar to the Fermi-level pinning effect, which limits the effective work function of Pt strip on a graphene to ~4.3 eV.

We found that this problem could be alleviated by using a low-temperature high-pressure hydrogen annealing process. Using this result, the doping profile of graphene under the metal strip could be controlled from 4.315 eV to 4.688 eV, and a complementary ternary device could be demonstrated. Figure 1a shows a schematic illustration of a ternary graphene field effect transistor (TGFET). The inset figure shows the schematic of a cross-sectional view of a TGFET, consisting of Al_2_O_3_/metal strip/graphene. Al_2_O_3_ was deposited over the graphene channel to improve the stability of the TGFET by blocking the water-related molecules from ambient condition. Figure 1b shows a scanning electron microscope (SEM) image of the graphene channel with the metal strip. Gold source/drain contacts were formed after the graphene channel patterning. An Al or Pt metal strip was formed in the middle of the graphene channel. As a result, a P-N-P or N-P-N junction profile is formed in the graphene channel, depending on the work function of the metal strip.

Figure 1c shows a representative resistance curve of a graphene channel with an Al strip. Two distinct Dirac points (V_Dirac,0_ and V_Dirac,1_) shown in Fig. 1c originate at the Dirac point of the p-type region and n-type region. The original and additional Dirac voltages (represented as V_Dirac,0_ and V_Dirac,1_, respectively) are the charge neutrality point of the graphene channel without and with the metal strip. The inset of Fig. 1c shows a representative band diagram for different gate bias regions. At zero bias, the graphene under the Al strip is doped as n-type, while the other channel regions are slightly doped in p-type, forming P-N-P doping regions. As the gate bias increases (or decreases), the channel doping will be converted to n-type (or p-type). During the transition, the channel resistance sharply increases at the Dirac point of the p-type (or n-type) region. As a result, the transfer characteristics of the TGFET with an Al strip show three distinct current levels (I_0_’, I_1_’, and I_2_’) as a function of gate bias, as shown in Fig. 1d. This curve shape is similar to that of the QDFET mentioned above, but the scaling of this device is only limited by the additional width of the Al strip.

Figure 1d also shows the transfer characteristics of TGFETs with a Pt strip. Since the work function of a Pt strip is higher than the Dirac point of a graphene channel, the graphene should be strongly doped in p-type, and V_Dirac,1_ is expected to be in the electron branch. However, both the Pt and Al strips show V_Dirac,1_ in the hole branch (Fig. 1d). The detail shape of I_d_-V_g_ curve near I_1_’ is primarily affected by the residual charge density in the graphene channel under the metal strip. When a residual charge density increases, the transfer curve near the I_1_’ shows more flat region. Thus, from the shape of I_d_-V_g_ curve near I_1_’ for Pt and Al strip, we can tell that the residual charge density under the Pt strip is higher than Al strip case. The effective work function of Pt is close to 4.36 eV in this case. The physical mechanism of unintended work function shift is not clearly understood yet, but we have developed a method to reduce this problem.

Among various annealing processes in different ambient conditions, only high-pressure annealing in hydrogen (20 atm at 300 °C for 2 h) was found to be effective in recovering the effective work function of Pt. The sign of V_Dirac,1_ is reversed after the hydrogen annealing, as shown in Fig. 2a, indicating that the effective work function of Pt is increased substantially. Interestingly, annealing under the same conditions in nitrogen ambient did not yield noticeable changes in the effective work function of Pt (Supplementary Fig. S1). Thus, the restoration of the work function seems to be related to the interaction between metal, graphene, and hydrogen. Interestingly, the same annealing treatment on the graphene devices with Al strip didn’t yield any substantial change. In fact, any changes after the annealing observed in the devices with the Al strip appear to be the impacts of thermal annealing.

Since the hydrogen annealing may affect the interface of graphene and SiO_2_ substrate, we checked the influence of high pressure hydrogen annealing on the devices that don’t have metal strip (Supplementary Fig. S2). The electrical characteristics of graphene FETs showed only a slight change in the V_Dirac_, which can be explained with the effects of thermal annealing. Thus, we can conclude that the Fermi-level pinning-like work function shift only occurred at the interface of graphene and a high-work-function metal, and it can only be alleviated by a high-pressure annealing in hydrogen.

The actual amount of the Fermi-level shift in graphene by the metal strip can be extracted by fitting the experimental data using a modified constant mobility model^32^. Assuming the graphene channel as a variable resistor, the doping profile of graphene can be represented by a series connection of two different variable resistances. Then, the total resistance of the graphene channel with a metal strip is represented by following equation:


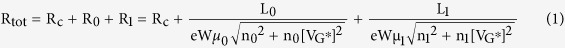


where R_c_ is the contact resistance; the resistance of the graphene beyond the metal strip is R_0_; the resistance of the graphene under the metal strip is R_1_; the carrier concentration in the graphene is 
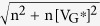
; L and W are the channel length and width, respectively; and μ is the mobility of graphene. Using this equation, the Dirac voltages (V_Dirac,0_, V_Dirac,1_) of graphene with/without metal strip were estimated simultaneously, and the corresponding effective work functions of metal were obtained. As shown in Fig. 2b, the effective work function can be represented by equation (2).


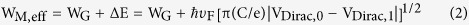


where W_M,eff_ is the effective work function of the metal strip, W_G_ is the work function of graphene (4.5 eV), and C/e is 2.4 × 10^11^ cm^−2^V^−1^. Figure 2c shows the change in the effective work function of Pt before and after nitrogen or hydrogen annealing. The effective work function was shifted by 34 meV after the nitrogen annealing and by 374 meV after the hydrogen annealing. As expected, the same high-pressure annealing did not affect the transfer characteristics of the TGFETs with an Al strip (Supplementary Fig. S3) as significantly as in the device with a Pt strip. The effective work function of Al was shifted by only 46 mV, which is close to the effect of thermal annealing in the Pt case.

We suppose that this improvement might be a result of the elimination of interface bonding through hydrogenation at the graphene/metal interface^33^. When the metal strip was formed on graphene, charge carriers were transferred between graphene and the metal strip. In the case of Pt whose work function is higher than that of graphene, the electrons moved to the metal side. Then, dipoles were generated at the interface of the metal and graphene. The direction of the dipoles were in a direction opposite to the work function of the metal, resulting in a decrease in work function, W_M,eff_ = W_M_−ΔV(d), where ΔV(d) is the potential change generated by the metal-graphene interaction, W_M,eff_ is the effective work function of metal on graphene, and W_M_ is the work function of the metal.

To further investigate the physical mechanism, the stability of effective work function modulation using hydrogen annealing was examined. Figure 3a shows the transfer characteristics of TGFETs measured at different temperatures. I_d_−V_g_ curves were measured with V_G_ = 0 V while the substrate temperature was changed from 25 °C to 125 °C with a step size of 25 °C. Since the drain currents at −10 V and +25 V are not significantly affected, the substantial temperature dependence appears to be related to the conductance change in the graphene under the Pt strip. In other words, the effective work function of Pt changes as a function of temperature. When this test was extended for 7200 s, similar stability characteristics were observed at even higher temperatures, as shown in Fig. 3b. V_Dirac,1_ was not significantly changed during operation up to 1,000 s. This indicates that the stability of an effective work function shift is more directly affected by the device operation temperature than the stress time.

These observations provide a clue to a possible mechanism that explains the effective work function shift after hydrogen annealing. Figure 3c shows the change in Dirac voltage (V_Dirac,0_ and V_Dirac,1_) and the activation energy extracted from the temperature dependence of the Dirac voltage change (see supplementary information). The activation energy could be extracted using the Arrhenius equation 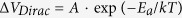. The activation energy decreased from 0.375 eV to 0.15 eV at temperatures above 75 °C. This change indicates there is another mechanism that reverses the effective work function increase by hydrogen annealing at higher temperatures.

Based on these observations, we tentatively concluded that the effective work function shift (i.e., V_Dirac,1_ shift) of Pt was caused by hydrogen diffusion into the interface of the graphene/Pt strip. The hydrogen molecules are adsorbed to the surface of Pt, forming Pt-H or Pt-OH complexes (Fig. 3d). These hydrogen bondings may deter the charge transfer process between Pt and graphene, and contribute to the restoration of the work function. Since the adsorption of hydrogen is not thermodynamically favorable at the low temperature ~300 °C, high-pressure annealing at 20 atm might have been necessary. In addition, as hydrogen bonding is not thermally stable, the effective work function of Pt decreased as the device operational temperature increased. The decrease in activation energy at higher temperatures may be attributed to enhanced hydrogen out-diffusion through the Pt layer. This rough model provides a consistent explanation for the experimental results, but the detailed mechanism should be studied in the future.

Since our primary interest is applying TGFETs to ternary logic architecture, the device characteristics of an n-type TGFET with an Al strip and p-type TGFET with a Pt strip are used to demonstrate complementary TGFETs and ternary inverter circuit, as shown in Fig. 4a. However, since the threshold voltage and gate dielectric of individual TGFETs were not fully optimized, the output characteristics of ternary inverters was modeled using the device parameters obtained from experimental devices.

Figure 4b shows the transfer characteristics of a standard ternary inverter consisting of only two ternary devices, simulated with an input voltage from 0 V to 2 V and V_DD_ = 2 V. The Dirac voltage of the p-type TGFETs was adjusted from 10 V to 30 V to balance the current level between the n-type and p-type TGFETs (Supplementary Fig. S4). With these adjustments, a reasonable working transfer curve for the standard TGFET inverter was obtained as shown in Fig. 4b. This result confirms that complementary TGFETs can be used for more general ternary logic circuits.

A unit ternary device showing three distinct current states was demonstrated using graphene FETs having P-N-P or N-P-N channel doping profiles. While the performances of these devices are not fully optimized, their feasibility for performing ternary logic functions was confirmed using experimentally extracted device parameters. This breakthrough will provide an important stepping-stone for future extreme-low-power electronics technology.

## Methods

### Methods Summary

A large sheet of single-layer graphene film grown by a chemical vapor deposition (CVD) method was prepared using a wet transfer process. After completing a back-gate graphene FET structure having a Pt source and drain, metal strips (Al, Pt) were inserted in the middle of the graphene channel. Then, the doping profile of the graphene channel was tuned to produce N-P-N and P-N-P profiles using high-pressure hydrogen annealing. The electrical characteristics of all devices were measured using a Keithley 4200 parameter analyzer.

### Graphene synthesis and transfer

A 1 cm × 1 cm monolayer of graphene sheet grown on Cu foil using a CVD process was transferred to 90-nm SiO_2_ thermally grown on a highly P-doped Si substrate using a poly (methyl methacrylate) (PMMA)-mediated transfer method^34^,^35^. When the transfer process was complete, the quality of graphene was assessed using Raman spectroscopy (Renishaw, λ = 248 nm, power = 20 mW). The Raman data of the graphene used in this work showed that the graphene is mostly a monolayer and that the integrated ratio of Raman peaks, I(D)/I(G), representing the quality of the graphene channel, was ~0.22 (Supplementary Fig. S5). The presence of a D peak indicates that the quality is reasonably good, but a considerable number of physical defects are still present.

### Fabrication of TGFET devices

For device fabrication, 20-nm Au was deposited on a graphene sheet and patterned using i-line contact photolithography and an etching process. Then, the graphene layer open to air was etched using an oxygen plasma process (process power = 50 W, process time = 90 s). After channel patterning using a Au hard mask, a 100-nm Au layer was deposited again using e-beam evaporation and patterned using i-line photolithography and an etching process. The Au hard-mask process was adopted to pattern both the channel and the source/drain region of graphene while minimizing the impact of photoresist residues in the graphene channel. The metal strip (Al, Pt of 10 nm) was formed in the middle of the graphene channel using i-line contact photolithography and a lift-off process. The surface of the graphene channel was passivated with 20-nm Al_2_O_3_ using an atomic layer deposition (ALD) process at 130 °C to improve the stability of devices, and annealed in ambient H_2_ at 300 °C for 2 h at 0 atm and 20 atm to control the doping profile of the graphene channel

## Additional Information

**How to cite this article**: Kim, Y. J. *et al*. Demonstration of Complementary Ternary Graphene Field-Effect Transistors. *Sci. Rep.* **6**, 39353; doi: 10.1038/srep39353 (2016).

**Publisher's note:** Springer Nature remains neutral with regard to jurisdictional claims in published maps and institutional affiliations.

## Supplementary Material

Supplementary Information

## Figures and Tables

**Figure 1:**
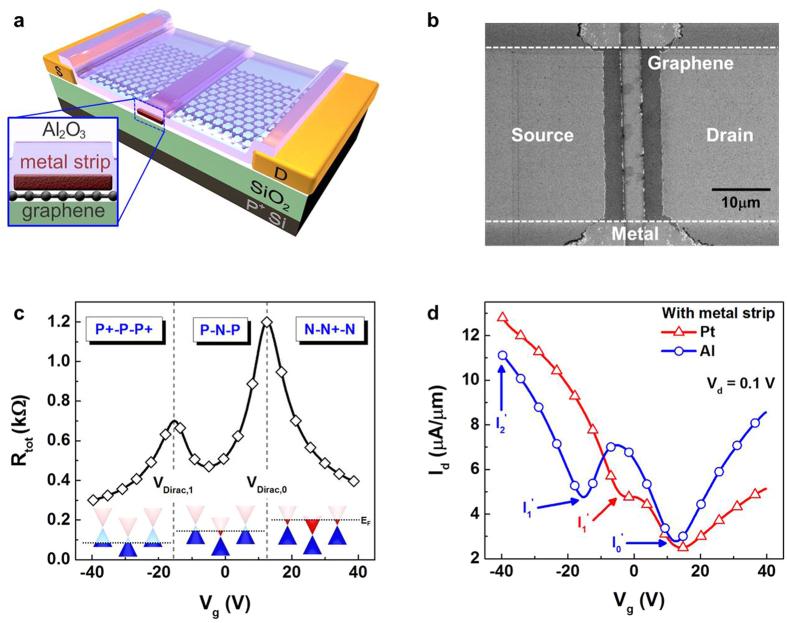
Structure and electrical characteristics of TGFETs. (**a**) Schematic of three-dimensional view of TGFETs. Inset figure shows the schematic of cross-sectional view of device. (**b**) SEM image of TGFETs. In this device, metal strips (Al, Pt) were inserted in the middle of the graphene channel to control the Fermi-level of graphene under the metal strip. (**c**) R_tot_−V_g_ characteristics of TGFETs with metal strip. This device shows additional Dirac voltage (V_Dirac,1_) with evidence of controlling the graphene channel using metal strip. Inset figure shows formation of P-N-P junction and changes in Fermi level of graphene and junction profile with gate voltage through band diagram of graphene. (**d**) I_d_−V_g_ curve of TGFET with different metal strips. Additional Dirac voltage appeared in hole branch of TGFETs with both Al and Pt strips.

**Figure 2:**
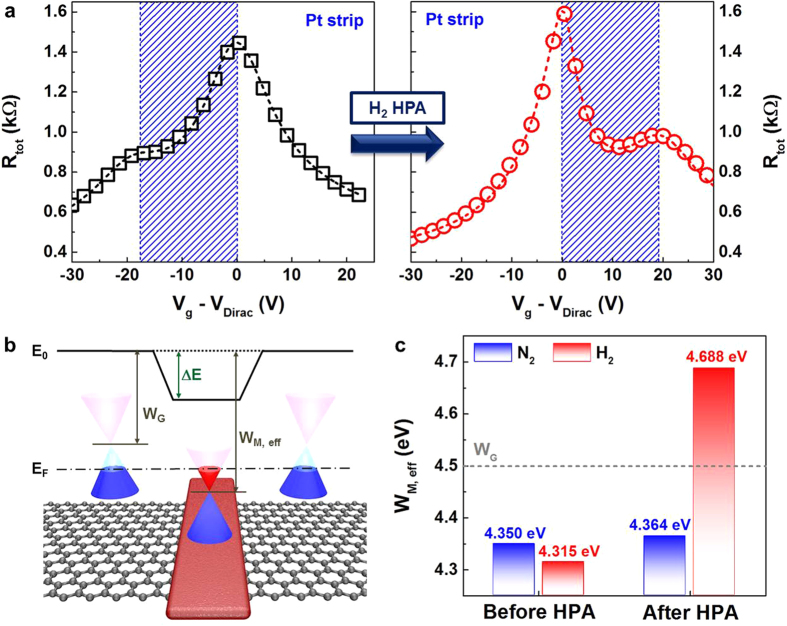
Controlling Fermi-level of graphene under metal strip with high-pressure hydrogen annealing. (**a**) R_tot_ (V_g_−V_Dirac_) characteristics of TGFETs with Pt strip. Left-hand figure shows electrical characteristics before high-pressure hydrogen annealing. Right-hand figure shows electrical characteristics after high-pressure hydrogen annealing. After high-pressure hydrogen annealing, additional Dirac voltage in this device transfers from hole to electron branch. This behavior indicates that the junction profile of graphene changes from P-N-P to N-P-N. In these figures, symbols indicate experimental results, and the dotted line indicates simulated results using equation (1). (**b**) Definition of effective work function of metal on graphene, represented by 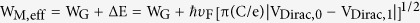. (**c**) Change in effective work function of Pt with different ambient conditions (N_2_ and H_2_). After high-pressure nitrogen annealing, the effective work function of Pt shifted from 4.350 eV to 4.364 eV. Effective work function of Pt shifted from 4.315 eV to 4.688 eV after high-pressure hydrogen annealing.

**Figure 3:**
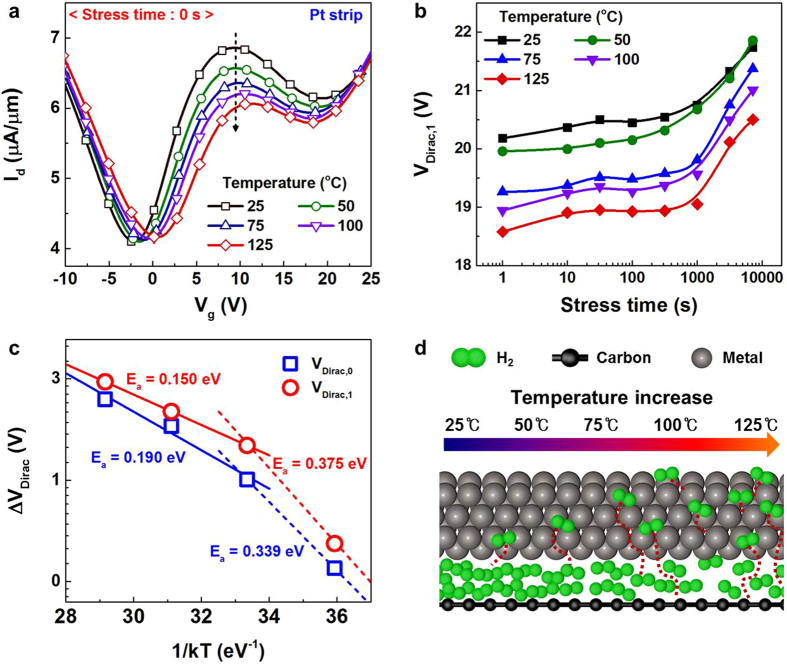
Temperature and time dependence of I_d_−V_g_ characteristics for TGFETs with Pt strip, and mechanism of junction profile change after high-pressure hydrogen annealing. (**a**) Temperature dependence of I_d_−V_g_ characteristics for TGFETs with Pt strip. (**b**) Time and temperature dependence of additional Dirac voltage (V_Dirac,1_), extracted from I_d_−V_g_ curve (**a**). I_d_−V_g_ was measured during stress time from 1 s to 10,000 s and with different temperatures from 25 °C to 125 °C. (**c**) Extraction of activation energy related to diffusion of hydrogen molecules. ΔV_Dirac_ satisfied Arrhenius plot, and activation energy decreased with increasing temperature based on 75 °C. (**d**) Schematic of diffusing hydrogen molecules with increasing temperature. Hydrogen molecules diffused from graphene/Pt strip interface, and this reaction accelerated after 75 °C.

**Figure 4:**
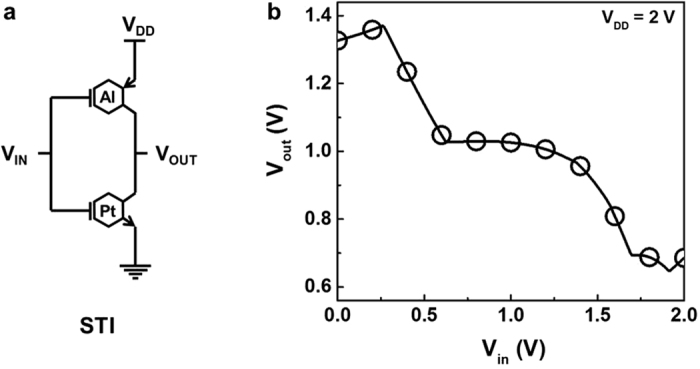
Circuits and simulated results of ternary inverter using TGFETs. (**a**) Circuits of ternary inverters, which are standard ternary inverters (STIs). Both n-type (Pt strip) and p-type (Al strip) TGFETs were connected in series. (**b**) Change in output voltage with input voltage of STI at V_DD_ = 2 V. In this research, characteristics of ternary inverter could be achieved using only two simple devices.
