# Supplementary material for: Demonstration of Complementary Ternary Graphene Field-Effect Transistors
Source: Sci Rep. 2016 Dec 19;6:39353. doi: 10.1038/srep39353 (PMC5172371; doi:10.1038/srep39353)
Supplement: Supplementary Information [file srep39353-s1.doc]

***Supplementary information***

***Demonstration of Complementary Ternary Graphene Field-Effect Transistors***

1Yun Ji Kim, 1So-Young Kim, 1Jinwoo Noh, 1Chang Hoo Shim, 1Ukjin Jung, 1Sang Kyung Lee, 1Kyoung Eun Chang, 2Chunhum Cho, and 1,2Byoung Hun Lee*

1Center for Emerging Electronic Devices and Systems, School of Materials Science and Engineering, Gwangju Institute of Science and Technology, Oryong-dong 1, Buk-gu, Gwangju, Korea 500-712

2Department of Nanobio Materials and Electronics, Gwangju Institute of Science and Technology, Oryong-dong 1, Buk-gu, Gwangju, Korea 500-712

Corresponding author: Byoung Hun Lee (bhl@gist.ac.kr)

**Table of Contents**

**IV characteristics of TGFET with nitrogen annealing (Figure S1) S3**

**Electrical characteristics of graphene FET with hydrogen annealing (Figure S2) S4**

**Electrical characteristics of TGFETs with Al strip (Figure S3) S5**

**Extraction of change in Dirac voltage (VDirac,1) S6**

**Simulated I-V characteristics of TGFETs (Figures S4) S7**

**Raman characteristic of graphene channel (Figure S5) S8**

**References S9**

**IV characteristics of TGFET with nitrogen annealing**


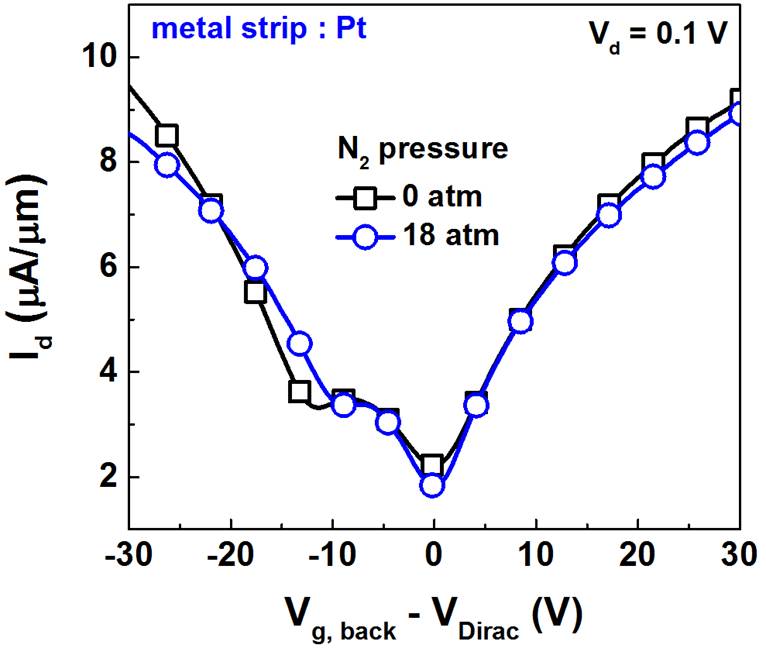


**Figure S1 Electrical characteristics of TGFET with nitrogen annealing.** Representative Id-V­g characteristics of TGFET with Pt strip with high pressure nitrogen annealing. Square symbol indicates Id-Vg characteristics at 0 atm, and circle symbol indicates Id-Vg characteristics at 18 atm. There are no significant changes in the additional Dirac voltage; the additional Dirac voltage appears as a hole branch with nitrogen annealing. This means there are no changes in junction profile at graphene under the metal strip after nitrogen annealing.

**Electrical characteristics of graphene FET with hydrogen annealing**


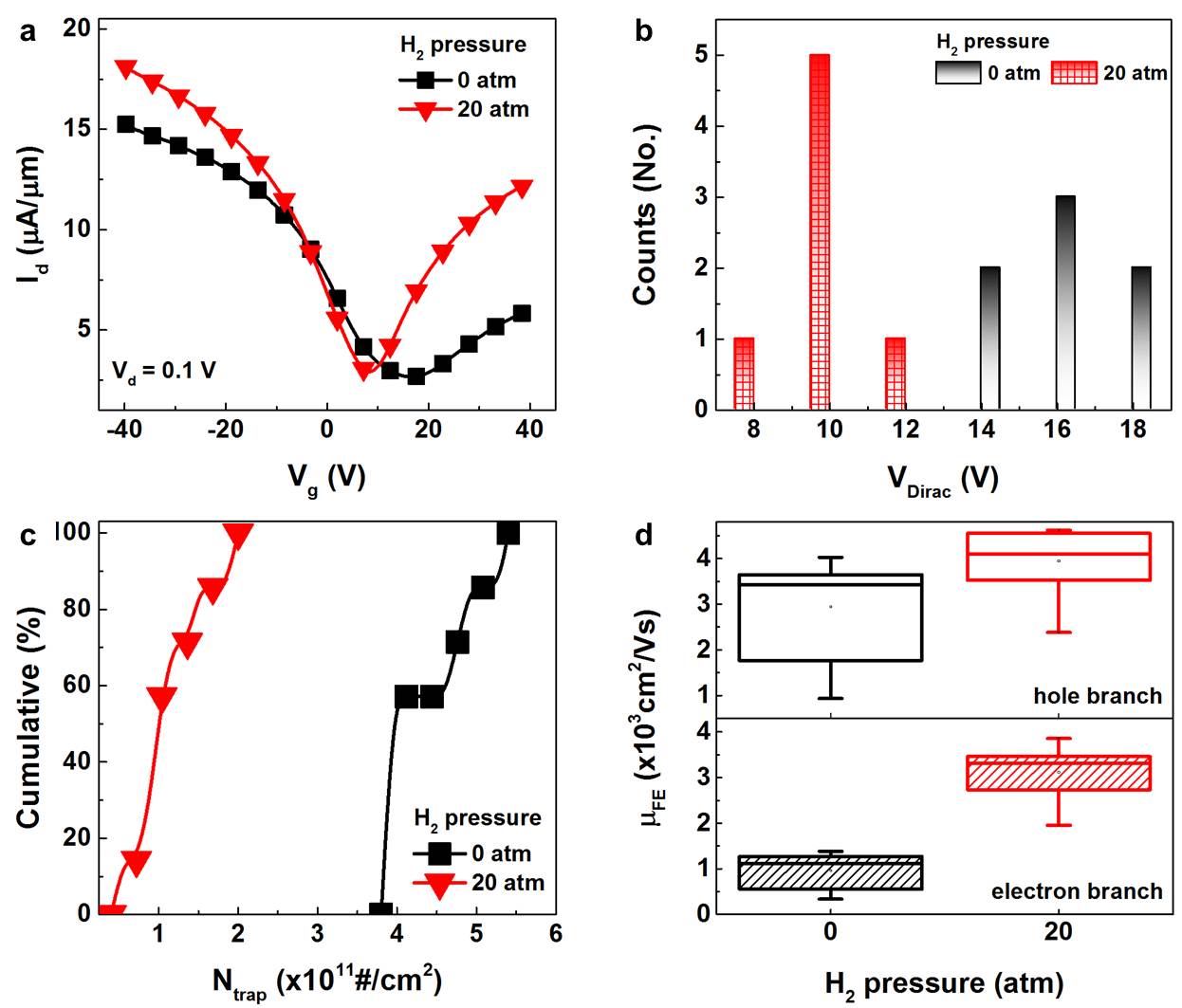


**Figure S2 Electrical characteristics of graphene FET with hydrogen annealing. a,** Representative Id-V­g characteristics of graphene FET after hydrogen annealing with different hydrogen pressures such as 0 and 20 atm. **b, c, d,** Change of Dirac voltage (b), trap density that is converted from hysteresis ($N_{trap}=\frac{C\cdot\Delta V_{Dirac}}{q}$) (c), field effect mobility, and (d) extracted from Id-Vg curve along the hydrogen pressure. The Dirac voltage decreases to 0 V, the trap density decreases drastically, and both electron and hole mobility increase with hydrogen annealing. These improvements to electrical characteristics indicate that the interface of graphene/SiO2 is improved through high-pressure hydrogen annealing.

**Electrical characteristics of TGFET with Al strip**


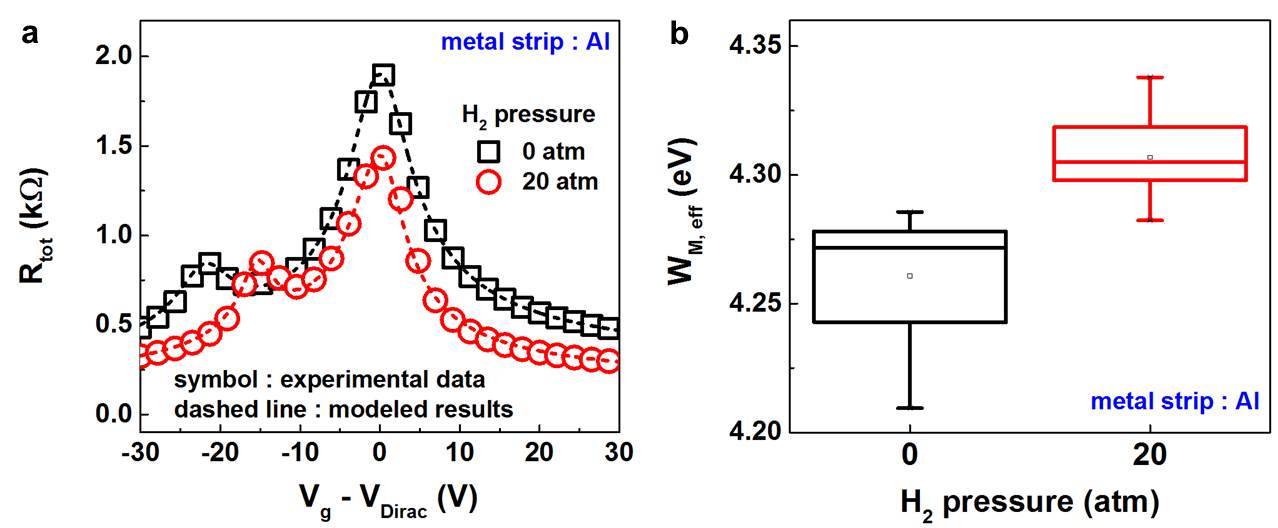


**Figure S3 Electrical characteristics of TGFET with Al strip. a,** Representative Rtot-Vg of TGFET with Al strip.Square symbol indicates Rtot-Vg characteristics when hydrogen pressure is 0 atm, and circle symbol indicates Rtot-Vg characteristics when hydrogen pressure is 20 atm. Dashed line is result of fitting data using modified constant mobility model described by Kim et al1. There are no significant changes at the additional Dirac voltage of TGFET with Al strip. Then, **b,** the shift of effective work function in TGFETs, was 46 mV for Al strip only.

**Extraction of change in Dirac voltage (VDirac,1)**

Parameters such as thermal degradation and hydrogen diffusion affect the changes in the Dirac voltage. To obtain the effect of hydrogen diffusion in the Id-Vg characteristics, the effect of thermal degradation should be excluded. For VDirac,1, the thermal degradation of VDirac,1 (VDirac,1[thermal])­ was estimated from the changes in VDirac,0, and the sole effect of hydrogen diffusion could be calculated by subtracting VDirac,1[thermal] from VDirac,1. Then, the change in VDirac,1 was expressed by ΔVDirac,1 ($\Delta V_{Dirac,1}=V_{Dirac,1}-V_{Dirac,1}\left[thermal\right]$).

**Simulated IV characteristics of TGFETs**


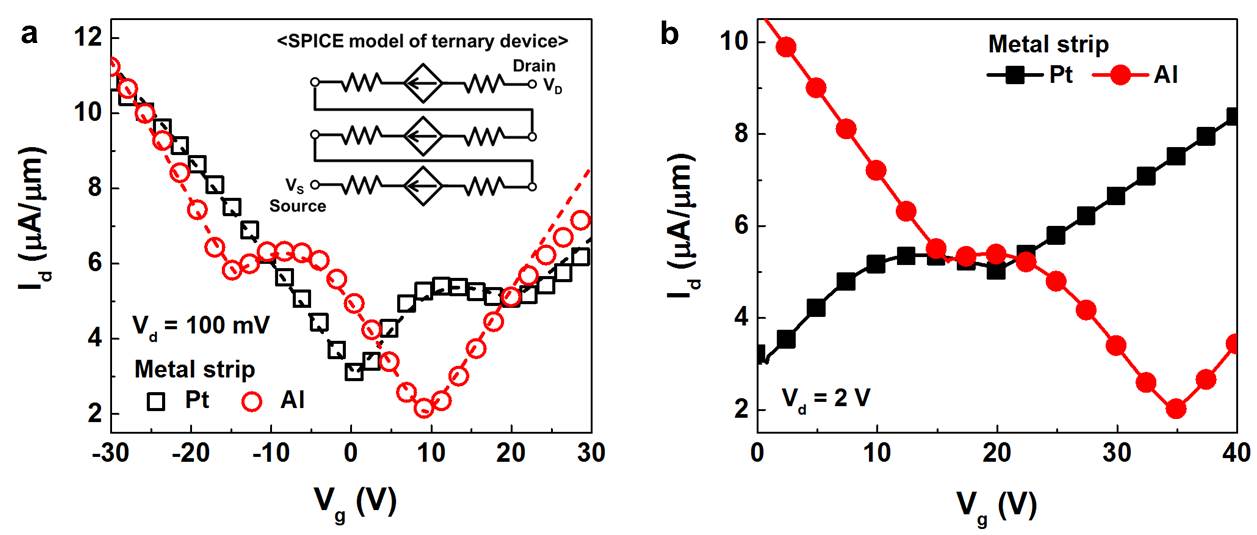


**Figure S4** **Simulated electrical characteristics of complementary TGFETs. a,** Simulated Id-Vg curve of TGFETs using SPICE model2. Symbol indicates experimental data, and dashed line is simulated data. Simulated data matches well with experimental data. Inset figure shows circuit diagram of TGFETs; three different types of graphene models are connected in series. **b,** Simulated Id-Vg­ curve of complementary TGFETs for adjusting to ternary inverter. To balance the current level between complementary TGFETs, Dirac voltage of p-type TGFET is shifted from 10 to 30 V.

**Raman characteristics of graphene channel**


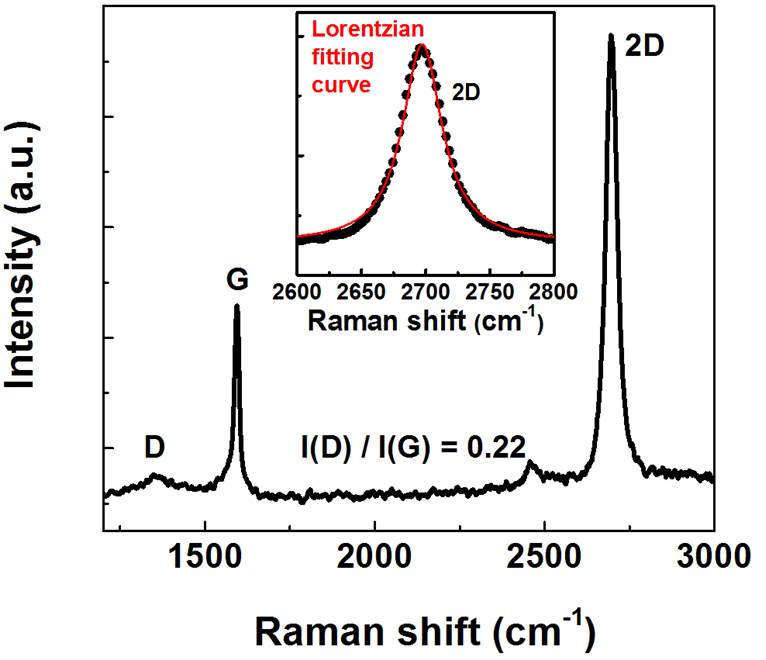


**Figure S5** **Result of measuring Raman spectroscopy on graphene channel.** After transferring graphene on the SiO2 substrate, the quality of graphene was measured by Raman spectroscopy. Insert figure shows 2D peak fitted by Lorentzian function; this means that single-layer graphene transferred well. Integrated area ratio of Raman peaks, I(D) / I(G), is approximately 0.22. This indicates that quality of transferred graphene is reasonably good.

**Reference**

1. Kim, S. *et al.* Realization of a high mobility dual-gated graphene field-effect transistor with Al2O3 dielectric. *Appl. Phys. Lett.* **94,** 62107 (2009).

2. Wang, H., Hsu, A., Kong, J., Antoniadis, D. A. & Palacios, T. Compact virtual-source current-voltage model for top- and back-gated graphene field-effect transistors. *IEEE Trans. Electron Devices* **58,** 1523–1533 (2011).
